# The diagnosis and treatment of latent tuberculosis by nurses in Brazil: a necessary strategy

**DOI:** 10.1590/0034-7167.2023760101

**Published:** 2022-11-28

**Authors:** Ricardo Alexandre Arcêncio, Pedro Fredemir Palha, Ethel Leonor Nóia Maciel

**Affiliations:** I Universidade de São Paulo, Escola de Enfermagem de Ribeirão Preto, Department of Maternal-Child Nursing and Public Health. Ribeirão Preto, São Paulo, Brazil; II Universidade Federal do Espírito Santo, Department of Nursing. Vitória, Espírito Santo, Brazil

Tuberculosis (TB) prevention, from preventing the progression of infection to active TB, is the main strategy to reduce its incidence and to reach the goals defined by the World Health Organization, according to the End TB strategy^(1)^.

Brazil, ranking 19^th^ in terms of the number of cases, assumed its commitment to institute preventive treatment, especially among People Living with the Human Immunodeficiency Virus (PLHIV), and ensuring that all people with a chance of developing active TB are covered by the strategy, which presents us with a great challenge. It is worth noting that, on the world stage, preventive treatment has been making timid progress since it was launched in 2018, and with the COVID-19 pandemic, there has been practically a stagnation, which worries health authorities around the world^(1)^.

Among the various international initiatives aimed at expanding the coverage of preventive treatment among vulnerable groups, there is the articulation and involvement of nurses through the case management strategy^(2)^. According to the Centers for Disease Control and Prevention (CDC), case management by nurses in the context of TB refers to follow-up of cases, both in active disease and in infection, from diagnosis, treatment to completion of therapy, in which the necessary resources for the control and elimination of TB are provided^(3)^.

In case management by nurses, all individuals with active disease become non-infectious upon completion of treatment, all individuals with TB infection remain non-infectious, and all individuals without TB infection TB do not be infected^(3)^. It is worth noting that there is consistent evidence of the effectiveness of case management by nurses in the context of TB and HIV comorbidities, by expanding care for vulnerable groups, better compliance, positive/favorable clinical outcomes, low risk of iatrogenic events, greater coverage of preventive treatment, equity and a people-centered approach^(2,4)^.

Nurses’ autonomy in TB diagnosis and treatment is revealed to be one of the main bets for advancing access to the health system and for overcoming geographic, economic, cultural and organizational barriers between people living with HIV^(2,4)^.

From a legal perspective, it is important to highlight a favorable environment for the intervention of case management by nurses, by investing in these professionals’ autonomy, whether for requesting diagnostic tests or prescribing treatments, according to Professional Practice Law 7.498/1986, which regulates the practice of nursing, the Resolution of the Federal Nursing Council (COFEN) 564/2017, which approves the Code of Ethics for Nursing Professionals, and Resolution (RDC) 471 of February 23, 2021, which provides for the criteria for prescription, dispensing, control, packaging and labeling of drugs based on substances classified as antimicrobials for prescription use, alone or in combination.

In the light of international policies, and from the perspective of the autonomy of nurses and their leadership, to reduce disparities and inequities in access to health services, the movement in Latin America and the Caribbean for Advanced Practice Nursing is essential for the inclusion of nurses in the prescription of preventive treatment for TB, as countries participating in the IMPAACT4TB project are already doing. In these places, the inclusion of shortened treatments, combined with the expansion for nurses’ prescription, was responsible for doubling the number of treatments offered in the services.

Considering that nursing is the largest contingent of human resources in the health system and that, in TB control services in Brazil, they are responsible for care management, the inclusion of nurses in the strategy of prescribing preventive treatment will make considerable progress towards achieving the goals of ending TB as a public health problem and alleviating suffering, illness and deaths resulting from TB.

It is time to take a step ahead of the disease, an opportune moment for the Ministry of Health to institute a strategic policy for TB prevention by nurses, supported by specific protocols and adequate qualification for the mission to eliminate TB in Brazil.

## References

[1] World Health Organization (WHO) (2021). Global tuberculosis report 2021 [Internet].

[2] Nyamathi A, Salem BE, Shin SS, Jones AA, Garfin DR, Yadav K (2021). Effect of a Nurse-Led Community Health Worker Intervention on Latent Tuberculosis Medication Completion Among Homeless Adults. Nurs Res.

[3] Centers for Disease Control and Prevention (CDC) (2012). Tuberculosis (TB). Menu of Suggested Provisions for State Tuberculosis Prevention and Control Laws. [Internet].

[4] Farley JE, Kelly AM, Reiser K, Brown M, Kub J, Davis JG (2014). Development and evaluation of a pilot nurse case management model to address multidrug-resistant tuberculosis (MDR-TB) and HIV in South Africa. PLoS One.

